# Stray Flux Sensor Core Impact on the Condition Monitoring of Electrical Machines

**DOI:** 10.3390/s20030749

**Published:** 2020-01-29

**Authors:** Pengfei Tian, Carlos A. Platero, Konstantinos N. Gyftakis, Jose Manuel Guerrero

**Affiliations:** 1 Department of Automatic Control, Electrical and Electronic Engineering and Industrial Informatics, Universidad Politécnica de Madrid, 28040 Madrid, Spain; tpf1213@gmail.com (P.T.); josemaguerrero2@hotmail.com (J.M.G.); 2 School of Engineering, The University of Edinburgh, Edinburgh EH8 9YL, UK; k.n.gyftakis@ieee.org

**Keywords:** electrical machine condition monitoring, field winding inter-turn fault, stray flux, stray flux sensor, stator inter-turn fault

## Abstract

The analysis of the stray flux for electrical machine condition monitoring is a very modern and active research topic. Thanks to this technique, it is possible to detect several types of failures, including stator and rotor inter-turn faults, broken rotor bars and mechanical faults, among others. The main advantages are that it involves a non-invasive technique and low-cost monitoring equipment. The standard practice is to use coreless flux sensors, with which the stray flux of the machine is not perturbed and there are no problems due to saturation or nonlinear behavior of the iron. However, the induced voltage in the coreless coil sensor may be very low and even, in some cases, have a similar amplitude to the noise floor. This paper studies the use of iron core stray flux sensors for condition monitoring of electrical machines. The main advantage of iron core flux sensors is that the measured electromotive force is stronger. In the case of large machines in noisy environments, this can be crucial. Two different types of iron core stray flux sensors and a coreless flux sensor are tested. A comparison of the three sensors is presented. Extensive experimental testing with all sensors shows the superiority and greater sensitivity of sensors with core versus the coreless ones.

## 1. Introduction

Detection of electrical machine faults via the stray flux has become a very modern and active research topic, as many early faults that create a magnetic asymmetry in the machines have become possible to detect [1]. 

In the case of induction machines, many different cases, such as broken rotor bars [2], inter-turn faults [3], problems in the bearings [4] and even the insulation’s health and ageing can be detected [5]. For these purposes, several techniques have been applied to analyze the stray flux, such as the bispectrum and covariance [6], the correlation coefficients [7] and the frequency extraction [8]. In some cases, two or more flux sensors are used [3,7,8]. The mechanical speed frequency also has an important role in rotor electrical and eccentricity fault detection [9]. Additionally, finite element method (FEM) studies have been performed studying the impact of different faults on the stray flux in induction machines [10,11].

In cases of permanent magnet synchronous machines, the stray flux has been used not only for fault detections [12] but also to measure the rotation speed [13].

Conventional rotor-wounded synchronous machines are the most important machines in power generation. The stray flux analysis may be a reliable method to detect early faults in such electrical machines. Past works suggest that inter-turn faults in both the rotor and the stator can be detected reliably [14,15].

Throughout the literature, the most frequently used flux sensors to measure the stray flux in electrical machines are coreless coils, normally known as research coils [1,2,4,5,6,7,8,9]. Some authors use rectangular-shaped coils [14,15]. Only a few authors use other types of sensors such as giant magneto resistive (GMR) [1] or tunneling magneto resistive sensors (TMR) [12,13].

The general operation principle dictates that the sensors supply an induced voltage, which is proportional to the derivative of the flux. The induced voltage is then recorded and analyzed with appropriate signal processing methods. The positioning of the sensor on the machine is also important as it can lead to the measurement of either the axial or the radial component of the stray flux.

Other industrial sensors, not often used for electrical machine monitoring, are the flux gate sensors or the hall effect sensor [16]. However, many researchers design their own sensors adapted to the size of the machine.

In other industrial applications the development of new magnetic field sensors is an ongoing research field. The use of microwires [17], the delta-E effect [18] and the magnetic flux leakage sensor [19] are some of the recent novelties in the area.

The most widespread sensor for electrical machine monitoring is the air core type, mainly for two reasons. This sensor type does not modify the stray flux of the machine, and the measurements are not influenced by the nonlinear behavior of the iron core due to the permeability, saturation, hysteresis or eddy currents. On the other hand, as the stray flux may have a low amplitude, the voltage induced in the sensor may also be small. Therefore, a large number of turns is required. 

In this paper, two different topologies of iron core stray flux sensors are presented and tested in the laboratory with a special synchronous machine. The magnetic core of the machine is modified by the iron of the sensor, and consequently the stray flux changes. The main advantage of the iron core sensors is that the induced voltage is significantly higher than in an air core sensor.

After a thorough experimental investigation, the conclusion is that the operation of the iron core stray flux sensor is appropriate, and the faults are detected more accurately than with conventional sensors.

## 2. Materials and Methods

### 2.1. Air and Iron Core Stray Flux Sensors

**Figure 1:**
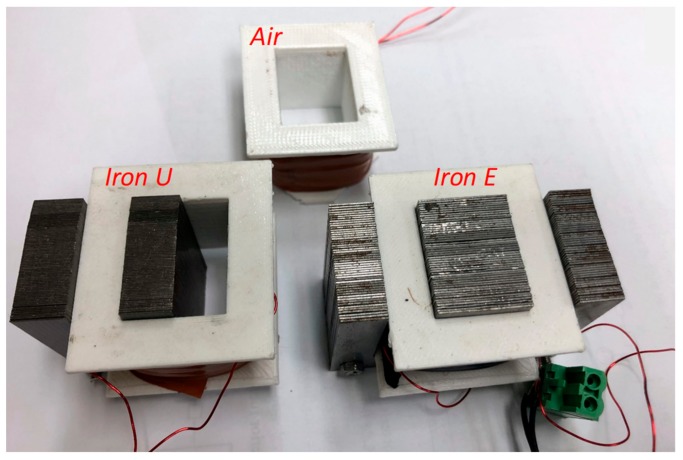
Flux sensors: Air, iron core U and iron core E.

The sensors used in this work were manufactured with a rectangular shape and comprise 200 turns of copper wire. The coils were placed in similar dielectric supports. Then at a second stage, the three individual coils were further discriminated by the use or not of an iron core. The first coil formed a coreless flux sensor. For the other two sensors, the coils were mounted on iron cores, with U and E shapes, respectively. The iron cores were built by standard magnetic steel laminations used in small transformers. The sensor coil supports are shown in Figure 1. 

**Figure 2:**
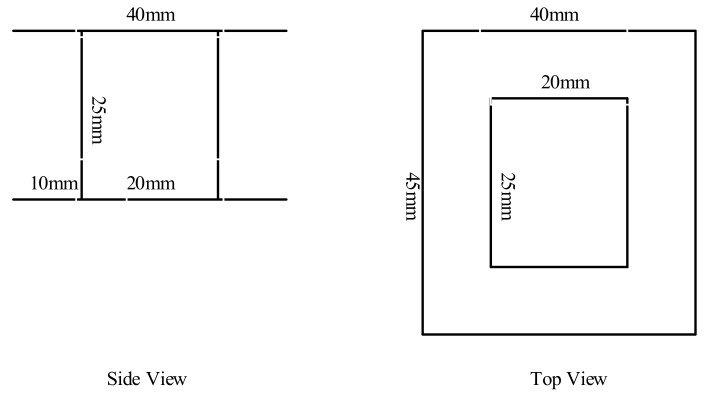
Side and top views of the coil support.

**Figure 3:**
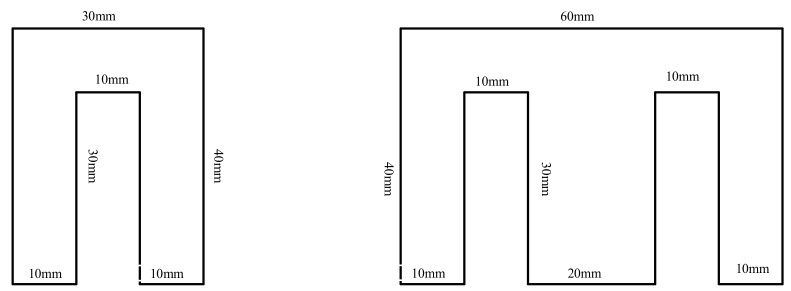
Dimension of magnetic steel lamination shape U and E.

The dimensions of the sensors are presented in Figure 2. The dimensions of the laminations are displayed in Figure 3. All the geometrical data of the sensors are presented in Table 1. The measurement system was just an oscilloscope which recorded the induced voltage in the sensors. Afterwards, the records were analyzed with MATLAB software in a computer.

The stray fluxes in the three sensors were different. In the case of the air core sensor, the stray flux was not distorted in any way. On the other hand, the iron cores increased the magnetic flux density due to the low permeability of the new flux path. Consequently, the sensors’ output voltage amplitude was significantly greater with iron core sensors.

### 2.2. Laboratory Experimental Setup

This section describes the experimental setup for testing the three stray flux sensors. The tests were performed in a specially reconfigured synchronous machine where it was possible to carry out different electrical faults in the rotor and the stator.

**Figure 4:**
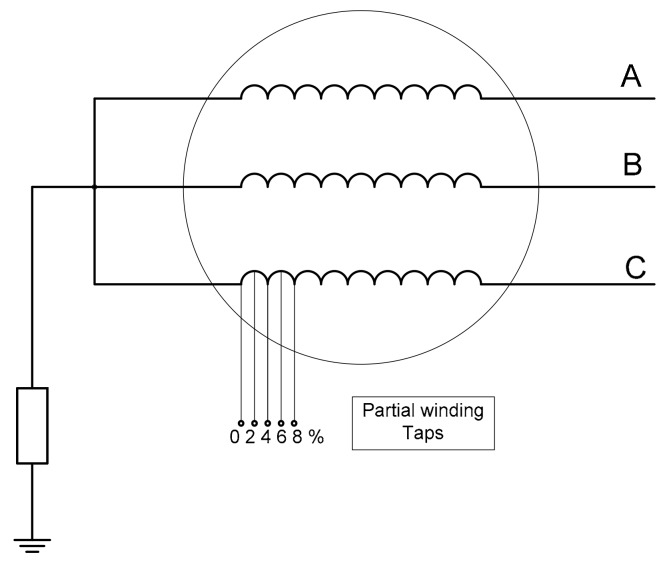
Synchronous machine stator connections.

**Figure 5:**
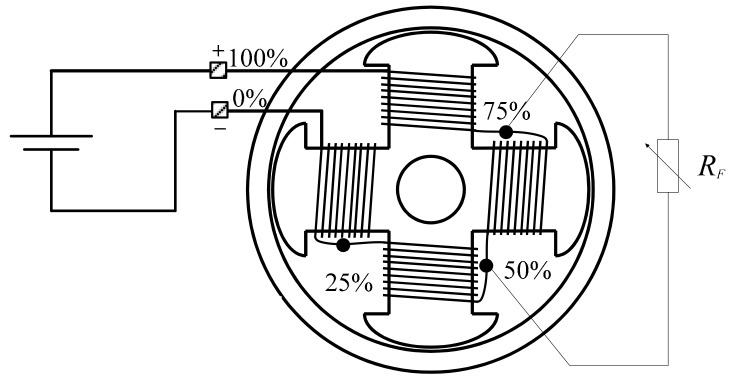
Synchronous machine rotor connections.

In Figure 4 and Figure 5, the electrical connection schemes of the stator and rotor are shown, respectively. The stator phase C has partial windings taps, which make it possible to perform inter-turn faults of variable severity in the stator (Figure 4). Additionally, the salient pole connections were reachable through a hole in the shaft, so inter-turn rotor faults of varied severity could be emulated by the use of the variable fault resistance R_F_ (Figure 5).

**Figure 6:**
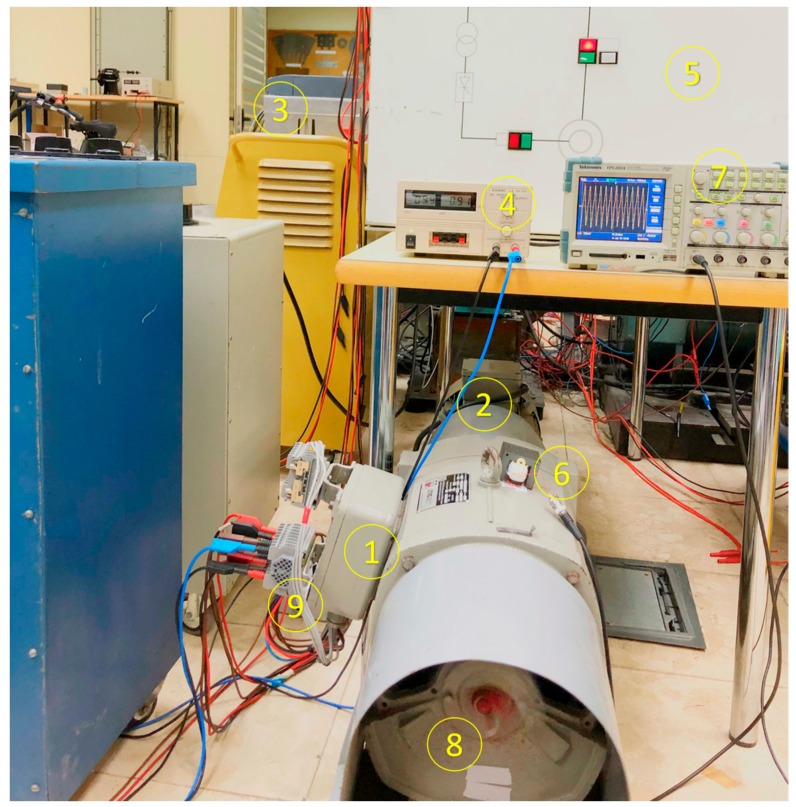
Experimental setup. (1) Synchronous machine, (2) asynchronous motor, (3) frequency converter, (4) DC-adjustable voltage source for excitation, (5) connection panel with synchronizer, (6) iron core E-type flux sensor, (7) oscilloscope, (8) pole connections and (9) stator tap terminals.

The complete experimental test bed is shown in Figure 6. The synchronous machine (1) was driven by an induction motor (2) fed by a frequency converter (3). The excitation of the machine was connected to an adjustable DC voltage source (4). The machine was connected to the power system by a panel (5) featuring a synchronizer. In this particular test, an iron core stray flux sensor with an E shape was placed in the upside part of the machine (6). Other tests were performed with the sensor placed in the right side of the machine. The induced voltage in the sensor was recorded in an oscilloscope (7).

As explained before, the pole connections were accessible in the shaft (8). In the connections, different resistances R_F_ were connected to emulate inter-turn faults. On the other hand, there were additional terminals with the taps of the stator phase C (9). The rated parameters of the special synchronous machine are shown in the Appendix A.

The synchronous machine was synchronized and connected to the grid. The active and reactive powers were controlled by the frequency converter (3) and the DC-adjustable voltage source (4), respectively.

## 3. Results

In this section, the results of the different tests under different conditions are presented. The first set of tests was performed with the healthy machine. The second set of tests corresponds to the machine with rotor inter-turn fault with different fault severities. This fault produced an imbalance related to the mechanical frequency, which in this case was 25 Hz. Finally, the third set of tests corresponds to inter-turn fault in the stator. This fault normally produces a signature at 850 Hz and 950 Hz. These frequencies (f_SISC_) are related to the electrical frequency (f_1_) multiplied by number of stator slots (k) divided by the pair poles (p), according to Equation (1). In this case, the stator slots were 36 and the pole pairs were 2, while the electrical frequency was 50 Hz.

(1) $$f_{SISC}=\;\left[\frac{k}{p}\pm 1\right]f_{1}$$

### 3.1. Healthy Condition

This section presents the results of the three sensors measuring stray flux in a laboratory synchronous machine in healthy conditions in two different positions, one in the upper part of the machine and the other in one side, as explained in the previous section.

**Figure 7:**
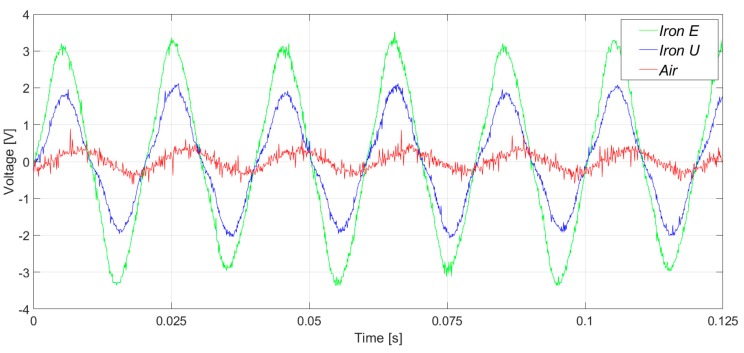
Induced voltage measurement in healthy conditions, in the right-side position.

**Figure 8:**
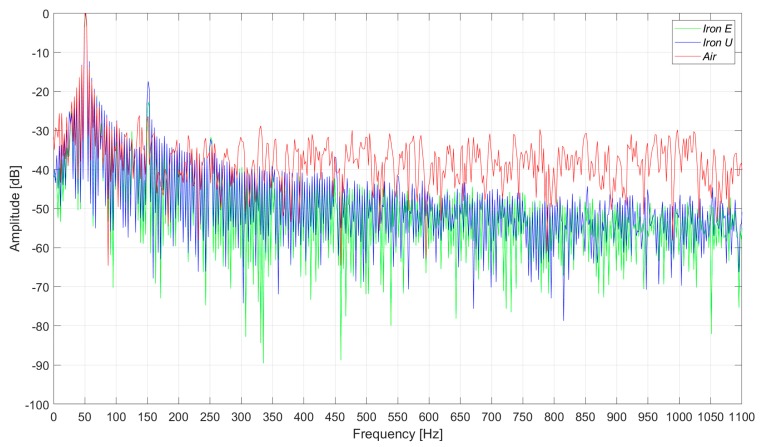
Frequency analysis of the induced voltage measurement in healthy conditions, in the right-side position.

In Figure 7, the induced voltages in the three flux sensors are presented. In the case of the healthy machine, the main component of the voltage corresponds to the 50 Hz frequency harmonic, as shown in Figure 8. As it can be clearly observed in Figure 7 and Figure 8, the signals and their respective harmonic components were similar. However, the voltage amplitudes offered by the iron core sensors were between four and seven times greater than those offered by the air core sensor. 

**Figure 9:**
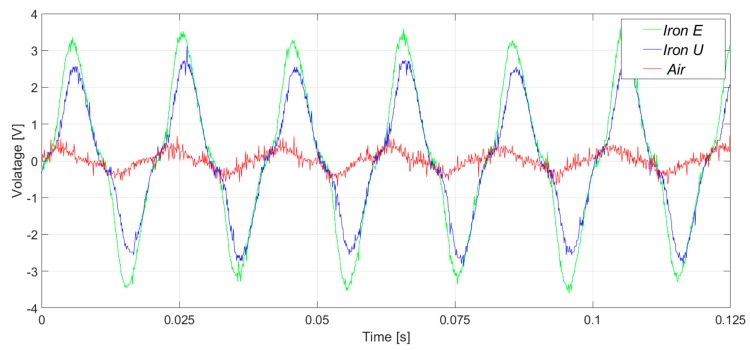
Induced voltage measurement in healthy conditions, in the upside position.

**Figure 10:**
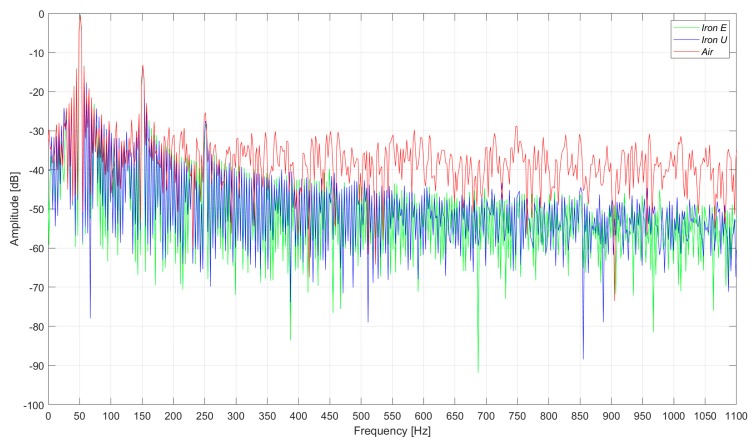
Frequency analysis of the induced voltage measurement in healthy conditions, in the upside position.

The measurements were taken in a laboratory where there were some electronic power converters in operation. Consequently, the three sensors had a high noise floor. However, the noise influenced the case of the air core sensor the most (Figure 9 and Figure 10). In Figure 9 and Figure 10, the induced voltages and the harmonics analysis are presented for the case of the healthy machine with the flux sensors placed in the upside position.

Table 2 shows the results obtained by the three sensors under healthy conditions at different positions. A comparison between the 50 Hz, the 25 Hz and the 850 Hz components are presented, as these frequencies are typical in the rotor and stator inter-turn faults.

### 3.2. Rotor Inter-Turn Faulty Conditions

This subsection presents the results from the three sensors measuring stray flux analysis in the case of rotor inter-turn faults with 5% and 15% of fault severity.

In the case of an inter-turn fault, one of the poles of the machine had a lower number of winding turns in operation. Consequently, a magnetic imbalance was created, as one of the poles produced a lower magnetomotive force than the others. Under such conditions, a harmonic corresponding to the rotating frequency is expected to appear in the stray flux of the stator [14,15].

**Figure 11:**
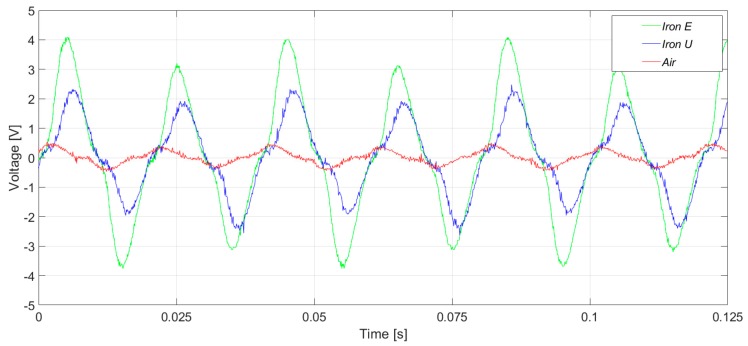
Induced voltage measurement with a 5% inter-turn rotor fault severity, in the upside position.

**Figure 12:**
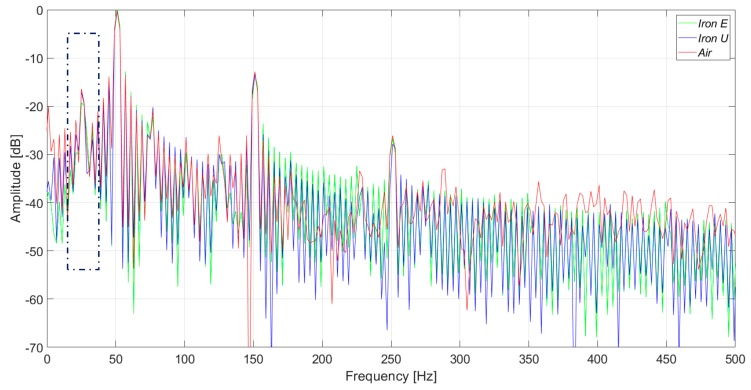
Frequency analysis of the induced voltage measurement with a 5% inter-turn rotor fault severity, in the upside position.

In Figure 11, the induced voltages recorded by the three sensors are presented for operation under 5% rotor inter-turn fault. While the machine had four poles, the rotating mechanical frequency was 25 Hz. The corresponding harmonic analysis is displayed in Figure 12 for all sensor cases.

**Figure 13:**
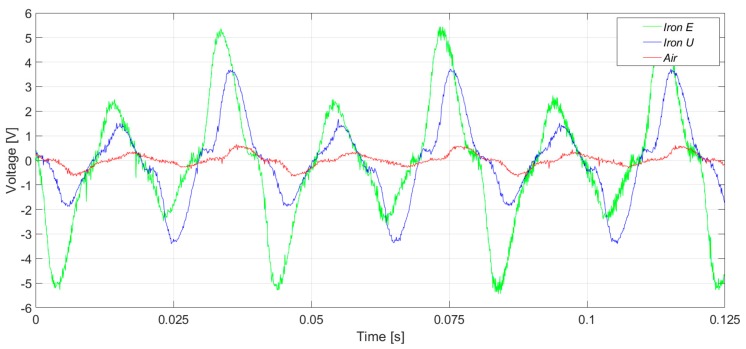
Induced voltage measurement with a 15% inter-turn rotor fault severity, in the upside position.

**Figure 14:**
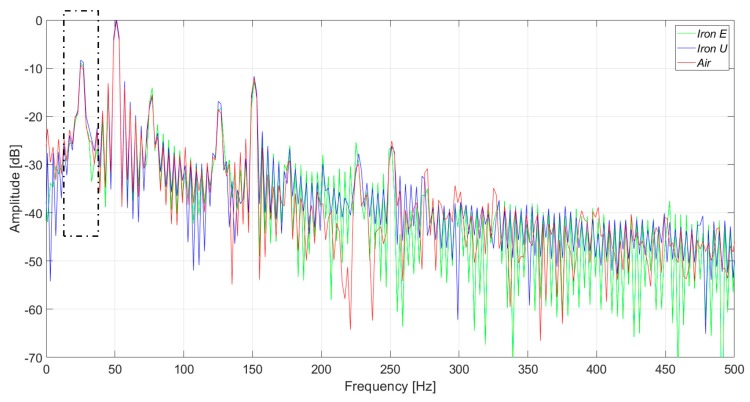
Frequency analysis of the induced voltage measurement with a 15% inter-turn rotor fault severity, in the upside position.

The induced voltage amplitude was greater in the case of iron core sensors than it was for air core sensors, especially in the E-shape type. Moreover, the calculated spectra were similar. It could be clearly seen that the 25 Hz harmonic, corresponding to the inter-turn fault in the field winding, presented an increased amplitude compared to the healthy machine. In Figure 13 and Figure 14, the induced voltage and the harmonics analysis in the three sensors are presented, respectively, for a 15% rotor inter-turn fault. The results were close to those presented above, but the amplitudes of the 25 Hz components were greater than in previously studied cases, as expected.

From Table 3, it can be clearly seen that the 25 Hz component could be detected by the three sensors. Furthermore, the amplitude differences between the fundamentals at 50 Hz and the 25 Hz signatures were similar. However, the induced voltage amplitude in the iron core E shape was eight time greater than in the case of the air core.

### 3.3. Stator Inter-Turn Fault Conditions

This section presents the results from the three sensors measuring stray flux analysis in a laboratory synchronous machine in the case of stator inter-turn faults. The severity level of the fault was set to 2% and 4% of the winding. In both cases, a 3 Ω limiting resistor was connected with the shortened loop in order to limit the developed short current of high amplitude, so that the machine was kept safe from overheating and catastrophic damage.

**Figure 15:**
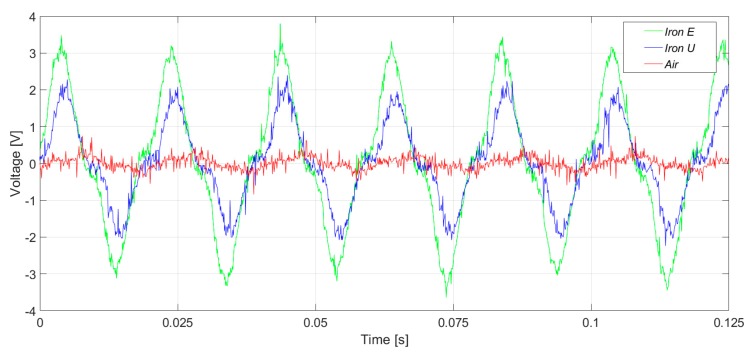
Induced voltage measurement with a 2% inter-turn stator fault severity, in the upside position.

**Figure 16:**
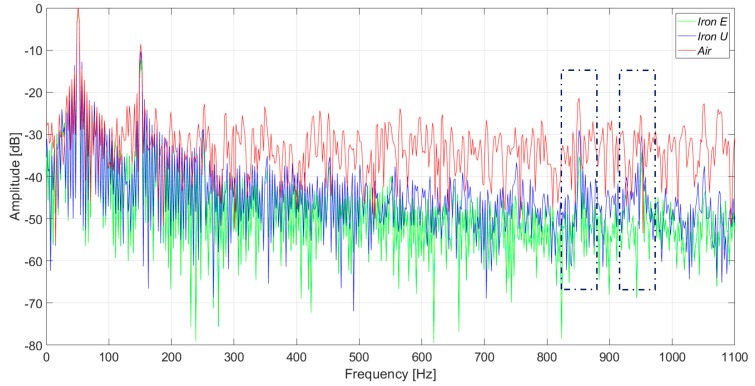
Frequency analysis of the induced voltage measurement with a 2% inter-turn stator fault severity, in the upside position.

Figure 15 and Figure 16 present the induced voltage waveforms in both the time domain and frequency domain under operation with 2% stator inter-turn fault. The synchronous machine tested had 36 stator slots and 2 pole pairs. Therefore, the 17th and the 19th harmonics were raised in the case of an inter-turn fault in the stator. In the case of iron core sensors, these harmonics were quite clear. However, the same harmonics were hidden in the noise floor for the case of the coreless sensor. 

**Figure 17:**
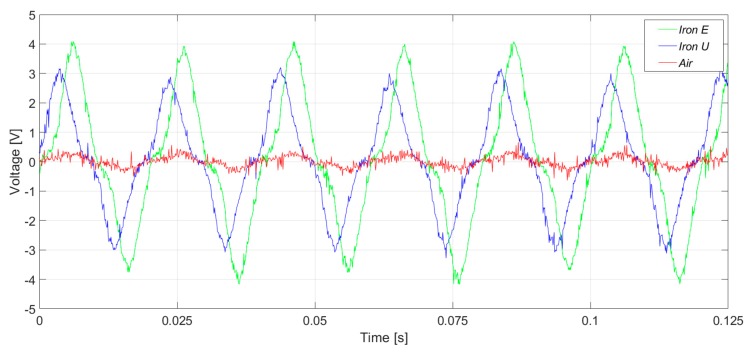
Induced voltage measurement with a 4% inter-turn stator fault severity.

**Figure 18:**
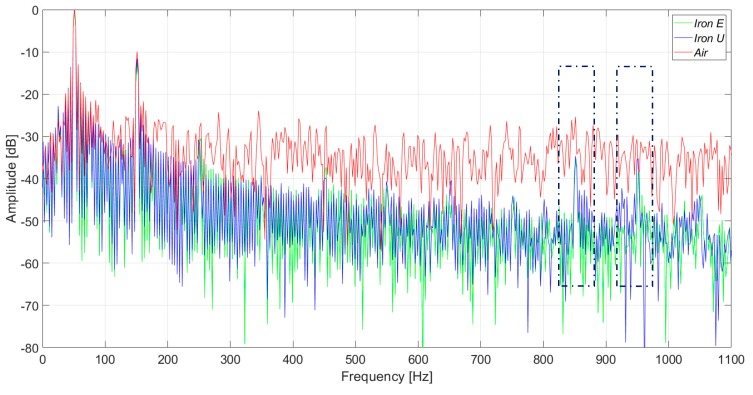
Frequency analysis of the induced voltage measurement with a 4% inter-turn stator fault severity.

In Figure 17 and Figure 18, the induced voltage waveforms and the respective harmonics analysis for the three sensors are presented, respectively, for machine operation with a 4% stator inter-turn fault. The results were close to those presented above. The amplitudes of the 17th and 19th harmonics components were similar to those obtained for 2% fault severity, and the non-monotonous behavior was similar to the case of the induction machines [20].

The results analyses for machine operation under stator inter-turn faults are summarized in Table 4 for all used sensors and fault severity levels. 

## 4. Discussion 

Air core sensors have been the usual solution in past works. The sensors consist of rigid coils with different shapes, either round or rectangular. The coils should have a high number of turns to increase the sensor’s sensitivity, since the stray flux amplitude is very low due to the high magnetic reluctance between the stator iron core and the exterior of the machine. As a result, only a small fraction of the flux radiates outside the machine.

In this paper, two different iron core stray flux sensors are presented and compared to an air core flux sensor with a similar number of turns. The iron core shapes used are U and E, typically found in small transformers. At the beginning of this research, two different problems were expected. On one hand, the iron core of the sensor would modify the magnetic circuit when placed close to the machine. Consequently, the stray flux in this part of the machine would be different than in the rest. It was not certain that the assessment of the flux under such conditions could identify the faults. After the tests, we can conclude that the iron core stray flux sensor can identify the faults in exactly the same way as the conventional coreless one.

On the other hand, the saturation and the nonlinear behavior of the iron can modify the induced voltage in the sensor coil. According to the performed tests, the waveforms in the iron core sensor and in the air core sensor are similar for frequencies up to 300 Hz. For frequencies above 300 Hz, the iron core sensors have lower gains. But in the case of stator inter-turn faults, the 17th and 19th harmonics are better detected than in the case of the air core sensor.

The use of iron presents an important advantage. The induced voltages in the coil are greater than in the case of the air core sensor. In the executed tests, the obtained amplitudes are between four and seven times higher. 

In Table 5, a summary of the results is presented. To evaluate the sensibility of the sensors, the amplitude differences of the various harmonics from their respective fundamentals are compared.

In the case of the E-shape sensor, if an inter-turn fault happens in the rotor, the amplitude of the 25 Hz harmonic increases by 9 dB (from −26 dB to −17 dB) for 5% and 19 dB (from −26 dB to −7dB) for 15% fault severity respectively. The obtained results by using the U shape are similar, increasing by 9 dB and 17 dB for 5% and 15% inter-turn rotor faults, respectively. For the air core sensor, the increases are 7 dB and 14 dB, respectively.

The inter-turn stator faults are undoubtedly better observed by using the iron core sensors. The amplitude increase of the 17th and 19th harmonics is remarkable when compared to the adjacent harmonics. This can be clearly seen in Figure 16 and Figure 18. On the other hand, these harmonics are not so easily detected when the air core sensor is applied.

## 5. Conclusions

The normal practice in electrical machine monitoring is the use of air core flux sensors. After being mounted outside of the monitored machine, the induced voltage is recorded and analyzed. In this way, the stray flux of the machine is not perturbed, and there are no problems due to saturation or nonlinear behavior of the iron. However, the induced voltage may be weak, mainly due to the high reluctance between the actual stator iron core and the sensors.

Instead, this paper proposes iron core stray flux sensors with E and U shapes for the same task. These sensors have been built with standard iron lamination used for small transformers. Although the magnetic circuit of the machine is slightly modified, as is the stray flux, the faults can be easily detected. The characteristic frequencies of the rotor and stator inter-turn fault can be clearly observed in the performed tests. On the other hand, the nonlinear behavior of the iron due to the permeability, saturation, hysteresis or eddy currents does not affect the fault detection.

Two different types of iron core stray flux sensors and an air core flux sensor were tested and compared. All sensors have the same geometry and number of turns in the rigid coil. The correct operation of the iron core stray flux sensors was validated by experimental results in a specially reconfigured synchronous machine where inter-turn faults were performed in the rotor and in the stator windings. 

The analysis results suggest that the use of iron core flux sensors is advantageous. The main advantage is a significantly greater amplitude of the induced voltage for the same stray flux. This feature can be crucial for the case of large machines in noisy environments. Furthermore, the sensitivity of the iron core flux sensors to the signatures related with stator inter-turn faults was significantly greater than that of air core ones. Finally, in the case of rotor inter-turn faults, the use of the iron core sensor with an E shape offered slightly better results.

## Figures and Tables

**Table 1:** Sensor data.

Type of Sensor	Iron E	Iron U	Air
Copper Turns	200	200	200
Box Length (mm)	45	45	45
Box Width (mm)	40	40	40
Box Height (mm)	25	25	25
Hollow Length (mm)	26	26	26
Hollow Width (mm)	20	20	20
Iron U Length (mm)		40	
Iron U Width (mm)		30	
Iron U Foot Width (mm)		10	
Iron E Length (mm)	40		
Iron E Width (mm)	60		
Iron E Right and Left Foot Width (mm)	10		
Iron E Middle Foot Width (mm)	20		

**Table 2:** Results of healthy condition.

Condition	Healthy Upside			Healthy Right Side		
Type of Sensor	Iron E	Iron U	Air	Iron E	Iron U	Air
Max Voltage [V] (Abs value)	3.6	3.12	0.824	3.52	2.88	0.888
Amplitude at 50 Hz [dB]	0	0	0	0	0	0
Amplitude at 25 Hz [dB]	−26	−26	−24	−25	−26	−23
Amplitude at 850 Hz [dB]	−46	−44	−32	−40	−44	−33

**Table 3:** Results of rotor inter-turn fault.

Condition	Rotor Inter-Turn 5%			Rotor Inter-Turn 15%		
Type of Sensor	Iron E	Iron U	Air	Iron E	Iron U	Air
Max Voltage [V] (Abs value)	4.08	2.64	0.56	5.68	3.76	0.72
Amplitude at 50 Hz [dB]	0	0	0	0	0	0
Amplitude at 25 Hz [dB]	−17	−17	−17	−7	−9	−10

**Table 4:** Results of stator inter-turn fault.

Condition	Stator Inter-Turn 2%			Stator Inter-Turn 4%		
Type of Sensor	Iron E	Iron U	Air	Iron E	Iron U	Air
Max Voltage [V] (Abs value)	3.8	2.4	0.84	4.32	3.56	0.664
Amplitude at 50 Hz [dB]	0	0	0	0	0	0
Amplitude at 850 Hz [dB]	−25	−29	−21	−36	−35	−25

**Table 5:** Summary of the Results.

Type of Sensor	Iron E	Iron U	Air
Healthy			
Amplitude [dB] (25 Hz)	−26	−26	−24
Rotor Inter-Turn 5%			
Amplitude [dB] (25 Hz)	−17	−17	−17
Rotor Inter-Turn 15%			
Amplitude [dB] (25 Hz)	−7	−9	−10
Healthy			
Amplitude [dB] (850 Hz)	−46	−44	−32
Stator Inter-Turn 2%			
Amplitude [dB] (850 Hz)	−25	−29	−21
Stator Inter-Turn 4%			
Amplitude [dB] (850 Hz)	−36	−35	−25

## Appendix A

**Table A1:** Rating of the synchronous machine.

Machine Type	3 Phase Synchronous Machine	10LCE-132ME1.4
Rated Power	3.4	kVA
Rated Voltage	400	V
Rated Power Factor	0.8	
Rated Frequency	50	Hz
Speed	1500	rpm
Rated Field Voltage/Current	32/2.9	V/A
Stator Slot	36	
Pole Pairs	2	
